# Benefits of extensive recruitment effort persist during follow-ups and are consistent across age group and survey method. The TRAILS study

**DOI:** 10.1186/1471-2288-12-93

**Published:** 2012-07-02

**Authors:** Esther Nederhof, Frederike Jörg, Dennis Raven, René Veenstra, Frank C Verhulst, Johan Ormel, Albertine J Oldehinkel

**Affiliations:** 1 Interdisciplinary Center Psychopathology and Emotion regulation, University Center for Psychiatry, University Medical Center Groningen, University of Groningen, Groningen, The Netherlands; 2 Friesland Mental Health Services, Leeuwarden, The Netherlands; 3 Department of Sociology, University of Groningen, Groningen, The Netherlands; 4 Department of Child and Adolescent Psychiatry, Erasmus University Medical Center, Rotterdam, The Netherlands

## Abstract

**Background:**

Extensive recruitment effort at baseline increases representativeness of study populations by decreasing non-response and associated bias. First, it is not known to what extent increased attrition occurs during subsequent measurement waves among subjects who were hard-to-recruit at baseline and what characteristics the hard-to-recruit dropouts have compared to the hard-to-recruit retainers. Second, it is unknown whether characteristics of hard-to-recruit responders in a prospective population based cohort study are similar across age group and survey method.

**Methods:**

First, we compared first wave (T1) easy-to-recruit with hard-to-recruit responders of the TRacking Adolescents’ Individual Lives Survey (TRAILS), a prospective population based cohort study of Dutch (pre)adolescents (at first wave: *n* = 2230, mean age = 11.09 (SD 0.56), 50.8% girls), with regard to response rates at subsequent measurement waves. Second, easy-to-recruit and hard-to-recruit participants at the fourth TRAILS measurement wave (*n* = 1881, mean age = 19.1 (SD 0.60), 52.3% girls) were compared with fourth wave non-responders and earlier stage drop-outs on family composition, socioeconomic position (SEP), intelligence (IQ), education, sociometric status, substance use, and psychopathology.

**Results:**

First, over 60% of the hard-to-recruit responders at the first wave were retained in the sample eight years later at the fourth measurement wave. Hard-to-recruit dropouts did not differ from hard-to-recruit retainers. Second, extensive recruitment efforts for the web based survey convinced a population of nineteen year olds with similar characteristics as the hard-to-recruit eleven year olds that were persuaded to participate in a school-based survey. Some characteristics associated with being hard-to-recruit (as compared to being easy-to-recruit) were more pronounced among non-responders, resembling the baseline situation (De Winter et al.2005).

**Conclusions:**

First, extensive recruitment effort at the first assessment wave of a prospective population based cohort study has long lasting positive effects. Second, characteristics of hard-to-recruit responders are largely consistent across age groups and survey methods.

## Background

The first purpose of the present study was to investigate if extensive recruitment efforts at the start of a prospective population based cohort study pay off in the long term. A large literature on the short term effects of extensive recruitment effort shows that such efforts can increase representativeness of the study population by decreasing non-response bias (see for instance Kessler et al. [1] or Nakash et al. [2]). However, it is not known to what extent increased attrition occurs during subsequent measurement waves among subjects who were hard-to-recruit and what characteristics the hard-to-recruit dropouts have compared to the hard-to-recruit retainers. The second purpose of the study was to investigate whether characteristics of hard-to-recruit participants vary depending on age of the sample and survey method. More specifically, does additional recruitment effort convince the same type of individuals in 11-year-old preadolescents who need parental consent to participate in a school-based survey as in 19-year-olds who do not need parental consent to participate in a web-based follow-up?

### Non-response

It is well known that non-response at baseline can lead to response bias in cohort studies. Non-responders are more frequently males, of lower socio-economic status, of non-western ethnicity, and have poorer academic achievement and more health problems than responders [3-6]. Although some researchers suggest that the effects of response bias are overestimated [7], others have shown that non-response at baseline is a threat for external validity [8].

### Recruitment effort

Different strategies have been described to reduce response bias, such as repeated mailings following initial non-response [9,10] and the use of alternative, shortened versions of measurement instruments [11]. In our own study, the TRacking Adolescents Individual Lives’ Survey (TRAILS), extra recruitment effort at the first measurement wave consisted of one or two house visits after no response to both an initial and a reminder letter had been received, and offering a two-month reflection period if the initial participation request was at an inconvenient time [5]. Different studies have shown that recruitment efforts lead to a more representative sample in terms of sex, age, race, socio-economic status and health [5,11,12]. Although the representativeness increases, the quality of the data has been shown to decrease with extra recruitment effort, because of more missing values and errors in data from late compared to early responders [10,12].

### Attrition

Attrition, or drop-out, is largely predicted by the same variables as non-response. Males [13,14], as well as participants with low socio-economic status [13], non-western ethnicity [13,15-17], low academic achievement [3,15,17,18] and physical and mental health problems [13,16-19] are particularly likely to drop-out from longitudinal studies. The observation that non-response is predicted by the same variables as attrition makes it plausible that participants for whom extra recruitment effort was done at inclusion are more likely to drop-out of longitudinal studies than those who were easy-to-recruit at inclusion. As far as we know, this has never been investigated. The first purpose of our study was to investigate how extensive recruitment effort at the first wave was related to attrition over an eight year follow-up period in the longitudinal study of adolescents TRAILS.

### Sample and survey characteristics related to success of extra recruitment effort

The second aim of our study was to identify factors that predicted attrition. Factors associated with non-response at the first wave (T1) have been described in detail by De Winter and colleagues [5]. At that time, the study population was about 11 years old and hence needed parental consent to participate in the study. The measurements took place at school. At the fourth assessment wave (T4), the study population was about 19 years old and did not need parental consent anymore, and a web-based survey method was used. Just like at T1, extra recruitment efforts were made at T4 to recruit initial non-responders. This gives us the opportunity to compare factors related to being easy or hard-to-recruit at these two assessment waves [5].

School-based surveys usually lead to higher response rates [13,14] compared to mail-based surveys, and obtaining written parental consent has been reported to be harder for boys, students with lower grades, students with non-Western ethnicity and less sociable children [20,21]. Age has also been associated with non-response and attrition. Adolescents and older adults are generally harder to include than children and young to middle-aged adults [4,6,11,17]. However, very little is known about how the effect of extensive recruitment efforts relate to sample and survey characteristics. In other words, the second purpose of this study was to investigate how extra recruitment effort in a web-based follow-up in 19-year olds affected attrition rates compared to extra recruitment effort in a school-based survey in 11-year olds in the same sample.

## Methods

### Participants

The TRacking Adolescents’ Individual Lives Survey (TRAILS) is a prospective cohort study of Dutch (pre)adolescents, with the aim to chart and explain the development of mental (ill)health from preadolescence into adulthood [22]. The present study involves data from all four assessment waves of TRAILS, which ran from March 2001 to July 2002 (T1), September 2003 to December 2004 (T2), September 2005 to August 2008 (T3), and October 2008 to September 2010 (T4), respectively. The study was approved by the Dutch Central Committee on Research Involving Human Subjects.

TRAILS participants were selected from five municipalities in the North of the Netherlands, including both urban and rural areas. Children born between 1 October 1989 and 30 September 1991 were eligible for inclusion, providing that their schools were willing to cooperate and that they met the study’s inclusion criteria [5]. Over 90% of the schools accommodating 2935 eligible children agreed to participate in the study.

Initially, 66% of parents and children agreed to participate (T1-easy-to-recruit). As parents were a source of information in TRAILS (see below), an ‘opt-in’ parental consent was necessary. Parents who refused to participate were asked permission to contact them again after 2 months, in order to minimise the number of refusals for temporary reasons. Parents with an unlisted telephone number were requested to contact the research team and pass on their number. If parents did not react to the initial letter, or to the reminder sent a few weeks later, a staff member paid a personal visit to their house. After two home visits, a letter was left with a reply card and a prepaid envelop. These extra recruitment efforts convinced 145 initial non-responders (T1-hard-to-recruit) and raised the final response rate to 76% (N = 2230, mean age = 11.09 years, SD = 0.56, 50.8% girls).

The extended efforts resulted in the recruitment of more vulnerable children and thus partially prevented a non-response bias regarding the prevalence of psychopathology [5]. Teacher reports, which were available for 40.7% of the non-responders, further revealed that the non-responders were more likely to be boys, to have a low socioeconomic background, and to perform poorly at school. Non-responders did not differ from responders regarding associations between sociodemographic variables and mental health outcomes [5].

Of the 2230 baseline participants, 96.4% (N = 2149, 51.0% girls) participated in the first follow-up assessment (T2). Mean age at T2 was 13.56 years (SD = 0.53). The response at the third wave was 81.4% (N = 1816, 52.3% girls). Mean age at T3 was 16.27 years (SD = 0.73). No extra efforts were undertaken to raise the response rates at T2 and T3.

At T4 the adolescents had reached the age of 18 or 19, and no parental consent was needed for participation anymore. At this wave, a custom research company (CRC) was hired to recruit and assess participants. The CRC was asked to recruit all respondents that had participated at T1 and at T2 or T3 and had not definitely refused further participation. The TRAILS research team sent information about the upcoming fourth wave, thereby explaining that the CRC would be responsible for the logistics. After participants had given informed consent, the CRC sent logon information for a web-based questionnaire. A gift certificate of 10 euro was included. Adolescents who did not respond to the questionnaire within 2–3 weeks, were contacted by telephone with the request to participate in (parts of) this wave. When they still did not respond after several reminders, or when adolescents could not be reached by telephone, a CRC employee paid one or two home visits, both announced and unannounced. The CRC realized a response rate of 72% (N = 1610). These responders are hereafter called ‘T4-easy-to-recruit’.

Participants who had not completed any assessments with the CRC, were contacted by the TRAILS research team. The TRAILS team approached these initial non-responders to evaluate the recruitment methods of the CRC, and to try to convince them to participate. The TRAILS research team also contacted T1 participants who had refused participation at both T2 and T3. Willingness to participate at T4 of initial non-responders was assessed and when they seemed willing, information about the fourth wave was sent, including a paper questionnaire and a gift certificate (10 Euro). The TRAILS team gave individuals who did not wish to fill out the full questionnaire the option to fill out a shortened version of the survey. The term web-based survey method should therefore be read as web or mail-based survey method throughout this paper. These extensive recruitment efforts lead to inclusion of 271 extra participants (T4-hard-to-recruit). The recruitment efforts increased the response rate of T4 to 84.3% (total *n* = 1881, mean age 19.1 (SD 0.60), 52.3% girls).

In short, T1-easy-to-recruit participants responded immediately; T1-hard-to-recruit-participants responded after several phone calls (until contact), one or two house visits and/or a two months reflection period. T4-easy-to-recruit participants responded to the CRC, which in some cases included reminders and one or two house visits; T4-hard-to-recruit participants responded only after the extra recruitment efforts of the TRAILS research team.

To be able to answer our second research question, we compared four groups: a) *T4-easy-to-recruit responders*; b) *T4-hard- to-recruit responders*; c) *T4-non-responders*, who participated in T3 but at T4 responded to neither the CRC nor the TRAILS team; and d) *drop-outs since T2 or T3*, who participated in T1 (and T2), but not in T3 and T4.

### Measures

TRAILS has biological, psychological, and social information from multiple sources, i.e. adolescents, their parents, their teachers and their peers. Huisman et al. gave an overview of all measurements of the first three waves [22]. The fourth wave was comparable to the earlier waves with a few adaptations. For example, a structured diagnostic interview [23-25] and a life stress interview [26] were administered; the Amsterdam Neuropsychological Tasks [27,28] were readministered; the adult version replaced the adolescent version of a number of questionnaires; and a number of age appropriate questions were added. For this paper, we used the following variables that we hypothesized to predict attrition:

#### Sociodemographic characteristics

Sociodemographic characteristics were assessed during an interview with one of the parents (usually the mother), administered at T1. The parent reported on whether the (biological) parents were divorced, the number of siblings, and whether the participant belonged to a single parent family. Educational level, occupational level [29] and socioeconomic position (SEP) [30] of the parents were also assessed at T1. Intelligence quotient (IQ) of TRAILS participants was estimated at T1 using the Vocabulary and Block Design subtests from the Revised Wechsler Intelligence Scales for children [27,31,32].

#### Educational level

The position in the educational system of all respondents at T2 and T3 was established by means of the so-called ‘educational ladder,’ developed by Bosker, Van der Velden, and Hofman [33]. This measure incorporates two aspects of a student’s position in the educational system, namely (1) the level of education (in the Dutch secondary educational system four tracks are distinguished corresponding to the level of difficulty), and (2) the progress within education. The scale ranges from 1 to 7 at T2 and 2 to 10 at T3. A score of 10 reflects the final exam of the highest track of secondary education. A score of 7 means that it will take three years until the final exam of the highest track can be obtained. Because the distances between the tracks can be considered as approximately similar, it is possible to scale them on an interval scale. Moving up a grade within the same track results in winning one point, whereas repeating a grade within the same track as well as streaming down to a lower track without repeating results in retaining the same score.

#### Sociometric status

Sociometric status of participants was assessed by means of peer nominations at T1 and T2. In classes with at least 10 TRAILS participants, children were asked to indicate whom they liked (peer acceptance), disliked (peer rejection), who bullied them (bullying), whom they bullied (victimization) and who helped them (helping). Children could nominate an unlimited number of same-gender and cross-gender classmates [34-37].

#### Substance use

Alcohol, cigarettes and cannabis use was assessed at T2, T3 and T4 by self-report questionnaires. Participants were asked to report whether they had ever used alcohol, cigarettes or cannabis (lifetime use), when they had started using it (age of onset) and the frequency of use. Although the validity and reliability of self-reports on substance use has been a subject of debate, previous research has concluded that, when anonymity is assured, self-report measures of substance use have acceptable validity and reliability [38,39].

#### Psychopathology

Externalizing and internalizing problems were assessed at T1, T2 and T3 by the Dutch version of the Child Behavior Checklist (CBCL) and the self-report version of this questionnaire, the Youth Self-Report [40,41]. At T4, the Adult Self-Report (ASR,[42]) was administered. These questionnaires contain a list of behavioural and emotional problems, which parents or the participant themselves can rate as 0 = not true, 1 = somewhat or sometimes true, or 2 = very or often true in the past 6 months. The broad-band dimension of Externalizing Problems encompasses the narrow-band scales Aggressive Behaviour and Rule-Breaking Behaviour. The dimension of Internalizing Problems included the scales Anxious/Depressed, Withdrawn/Depressed, and Somatic Complaints [41]. A Total Problem Score scale was constructed as the sum of all problem behaviours, that is, internalizing and externalizing problems as well as thought problems, attention problems and social problems.

Additionally, the Composite International Diagnostic Interview (CIDI, [23-25]) was administered at T4. The CIDI is a comprehensive, fully-structured interview designed to be used by trained lay interviewers for the assessment of mental disorders according to the definitions and criteria of ICD-10 and DSM-IV. It is intended for use in epidemiological and cross-cultural studies as well as for clinical and research purposes. The diagnostic section of the interview is based on the World Health Organization's CIDI [23-25]. Diagnoses were grouped into internalizing behaviour diagnoses, including anxiety and depressive disorders; and externalizing behaviour diagnoses, including substance abuse, conduct disorder and oppositional defiant disorder. A sum score of total problem behaviour diagnoses was calculated, including all internalizing and externalizing behaviour diagnoses, bipolar disorders and attention deficit hyperactivity disorder.

### Data analysis

To investigate whether the extra recruitment effort at T1 had a long-lasting effect, we used a logistic regression analysis with ‘being hard-to-recruit at T1’ as independent variable predicting response in the following measurement waves. To find out whether T1-hard-to-recruit-retainers (those that stayed in the cohort) were different from the T1-hard-to-recruit-dropouts (those that dropped out at T2,T3 or T4), the T1-easy-to-recruit- retainers or the T1-easy-to-recruit-dropouts, we performed single and multivariate multinomial regression analyses to provide estimates (odds ratio’s, including 95% confidence intervals) of the included predictors for each of the following categories: T4-responders that were T1-hard-to-recruit (‘T1-hard-to-recruit retainers’), T4-non-responders that were T1-hard-to-recruit (‘T1-hard-to-recruit dropouts’), T4-responders that were T1-easy-to-recruit and T4-non-responders that were T1-easy-to-recruit. To be able to show differences between T1-hard-to-recruit retainers and T1-hard-to-recruit dropouts, the T1-hard-to-recruit retainers were used as reference category, rather than the T1-easy-to-recruit retainers, which is the largest group. The following predictors were included in both the single and multivariate analyses: family composition, SEP, IQ, education, sociometric status, substance use, and psychopathology. The multivariate models were constructed using backward stepwise selection using likelihood ratio tests. P values were set at 0.1 to prevent relevant predictors from being excluded from the final model. Non-nested models (eg. when comparing the effects of parental education with a composite measure for socioeconomic status, which also includes parental education) were evaluated using Akaike’s (AIC) and Bayesian (BIC) information criteria.

For our second research question, we first used single multinomial regression analysis to provide estimates (odds ratio’s, including 95% confidence intervals) of the included predictors for each of the following categories: T4-easy-to-recruit, T4-hard-to-recruit, T4-non-responders and drop-outs since T2 or T3. Included predictors are family composition, SEP, IQ, education, sociometric status, substance use, and psychopathology. For predictors that were measured at T4 only, binary logistic regression was used. Then, to find out which predictors related most strongly to participation at T4, we performed a stepwise multivariate multinomial regression analysis using the same method as described above. In addition, we investigated possible interaction effects of predictors and T1 recruitment status on participation at T4.

The reporting of this observational study followed guidelines from the STROBE statement [43].

## Results

An overview of sample characteristics at each of the four measurement waves can be found in Table 1. At eight year follow-up, the response rate was 84%. With an initial response rate of 76%, this implies that 64% of the eligible children still participated in TRAILS eight years later.

**Table 1:** Characteristics of the sample at the four measurements waves of TRAILS

	** T1**	** T2**	** T3**	** T4**
*n*	2,230	2,149	1,816^c^	1,881^d^
mean age (*SD*)	11.09 (0.56)	13.56 (0.53)	16.27 (0.73)	19.1 (0.60)
% girls	50.8	51	52.3	52.3
response rate (%)	76^a^	96.4^b^	81.4^b^	84.3^b^

^a^ Of the 2,935 eligible children asked to participate at T1.

^b^ Of the 2,230 included children at T1.

^c^ Non-responders at T3 include 2 deceased, 7 who were physically or psychologically unable to participate, 4 who were detained or moved abroad, and 31 untraceable or unreachable participants. Other non-responders refused participation or did not return any information (*n* = 372).

^d^ Non-responders at T4 include 5 deceased, 3 who were physically or psychologically unable to participate, and 1 detained participant, 16 untraceable and 43 unreachable participants, and 9 participants who moved abroad. Other non-responders refused participation or did not return any information (*n* = 272).

### Effects of extensive recruitment efforts eight years later

**Figure 1:**
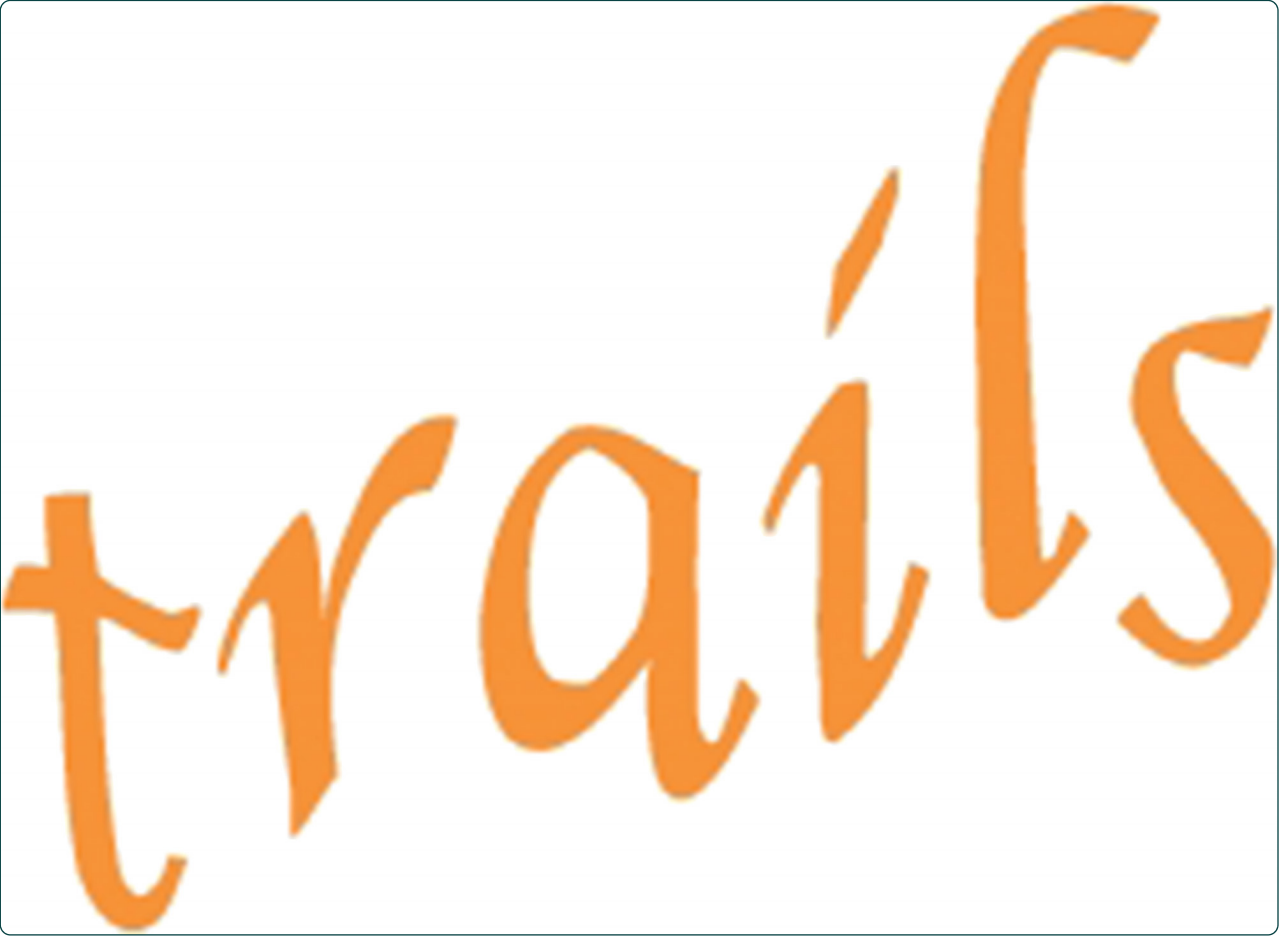
Participation trajectories of adolescents who were easy or hard-to-recruit at the first TRAILS assessment wave.

The first question in the present study was whether extensive recruitment effort at the first assessment wave (age 11) resulted in a more diverse sample eight years later, during the fourth assessment wave (age 19). Table 2 shows the response rates at T2, T3 and T4 of T1-easy-to-recruit responders and T1-hard-to-recruit responders, respectively. Of the T1-hard-to-recruit responders, 61% were still in the cohort at T4. As expected, attrition rates were significantly higher among T1-hard-to-recruit participants than among T1-easy-to-recruit participants, at all successive measurement waves (Table 2). This notwithstanding, over half of T1-hard-to-recruit participants were easy-to-recruit at T4 (Figure 1). Among the T1-hard-to-recruit participants we found no significant differences at T4 between retainers and drop-outs in sociodemographic variables, peer status or psychiatric symptoms (Table 3). This indicates no selective attrition of the most vulnerable T1-hard-to-recruit participants along the four measurement waves. In addition, T1-hard-to-recruit-retainers differ significantly from T1-easy-to-recruit retainers, indicating that the increased generalisability that was generated by the extra recruitment efforts at T1 is maintained throughout the waves.

**Table 2:** Response rates throughout the four measurement waves (T1-T4) of the TRAILS study for participants who were easy and hard-to-recruit at the first wave

	**T1**	**T2**		**T3**		**T4**	
	**n**	**n**	**%**	**n**	**%**	**n**	**%**
T1-easy-to-recruit	2,085	2,015	96.6	1,730	83.0	1,792	85.9
T1-hard-to-recruit	145	134	92.4	86	59.3	89	61.4
		OR	95% CI	OR	95% CI	OR	95% CI
T1-hard-to-recruit^a^		0.42*	0.22 – 0.82	0.30*	0.21 – 0.43	0.26*	0.18 – 0.37

*p < .05; Odds ratios (OR) and 95% confidence intervals (CI) from single binary logistic regression analyses predicting response.

^a^ T1-easy-to-recruit is the reference category; recruitment status is the independent variable.

**Table 3:** Multivariate multinomial logistic regression analysis predicting T1-hard-to-recruit-retainers, T1-easy-to-recruit-retainers, T1-easy-to-recruit-dropouts and T1-hard-to-recruit-dropouts. Retention and dropout observed at T4

	**T1-easy-to-recruit-retainer**			**T1-hard-to-recruit-retainer (ref.)**	**T1-easy-to-recruit-dropout**			**T1-hard-to-recruit-dropout**		
	**(*n* = 1,549)**			**(*n* = 55)**	**(*n* = 222)**			**(*n* = 33)**		
	**n(%)**	**OR**	**95% CI**	**n(%)**	**n(%)**	**OR**	**95% CI**	**n(%)**	**OR**	**95% CI**
*T1 Sociodemo-graphics*										
Non-western immigrant	102 (6.6)	0.40*	0.19-0.86	10 (18.2)	40 (18.0)	1.03	0.47-2.29	14 (42.4)	2.72	0.98-7.58
*(univariate effects)*		0.32*	*0.16-0.65*			*0.99*	*0.46-2.13*		3.32*	*1.25-8.77*
Lower education mother (≤lower tracks of sec. educ.)	491 (31.7)	0.44*	0.24-0.79	33 (60.0)	132 (59.5)	1.06	0.56-2.00	22 (66.7)	1.25	0.48-3.24
*(univariate effects)*		0.31*	*0.18-0.54*			*0.98*	*0.54-1.79*		*1.33*	*0.54-3.29*
Low family income (<€1,135)	183 (11.8)	0.48*	0.25-0.91	17 (30.9)	60 (27.0)	0.83	0.42-1.65	15 (45.5)	1.35	0.51-3.57
*(univariate effects)*		0.30*	*0.17-0.54*			*0.83*	*0.44-1.58*		*1.86*	*0.76-4.55*
Low IQ (wisc < 85)	213 (13.8)	0.55	0.30-1.03	18 (32.7)	65 (29.3)	0.88	0.45-1.73	13 (39.4)	1.08	0.41-2.81
*(univariate effects)*		0.33*	*0.18-0.59*			*0.85*	*0.45-1.60*		*1.34*	*0.55-3.28*
	mean (*SD*)	OR	95% CI	mean (*SD*)	mean (*SD*)	OR	95% CI	mean (*SD*)	OR	95% CI
*T1 Psychopathology*										
CBCL Internalizing problems	0.25 (0.19)	1.33	0.96-1.84	0.24 (0.17)	0.25 (0.20)	1.07	0.76-1.52	0.30 (0.24)	1.34	0.84-2.16
*(univariate effects)*		*1.00*	*0.76-1.31*			*1.03*	*0.76-1.38*		*1.26*	*0.85-1.86*
CBCL Externalizing problems	0.23 (0.19)	0.70*	0.53-0.91	0.31 (0.27)	0.30 (0.26)	0.95	0.71-1.27	0.28 (0.23)	0.74	0.47-1.17
*(univariate effects)*		0.71*	*0.56-0.89*			*0.97*	*0.76-1.23*		*0.89*	*0.61-1.30*

*p < .05; Odds ratios (OR) and 95% confidence intervals (CI) from multivariate multinomial logistic regression analysis; CBCL = Child Behavior Checklist (parent report); CBCL scores standardized; univariate effects of single multinomial regression analyses italicized. Nagelkerke *R²* = 0.145.

### Effects of extensive recruitment efforts at age 19

#### Sociodemographic characteristics

Table 4 shows sociodemographic variables and outcome measures for the 4 groups (T4-easy-to-recruit, T4-hard-to-recruit, T4-non-responders, and drop-outs since T2 or T3). Similar to T1 [5], T4-hard-to-recruit responders seem a relatively vulnerable group of adolescents: like T4-non-responders and T2/T3-drop-outs, they had a lower IQ, their parents were more often divorced, and they more often came from families with a low socioeconomic position. This suggests that extensive recruitment efforts to prevent attrition at age 19 increased the representativeness of our sample, like it did eight years earlier. Like at T1 [5], the socioeconomic position of T4-non-responders and T2/T3-drop-outs was lower than the socioeconomic position of the T4-hard-to-recruit responders (Table 4). Regarding IQ and parental divorce, drop-outs since T2 or T3 were equally likely to have a low IQ or divorced parents as T4-hard-to-recruit participants, while T4 non-responders were more likely to have a low IQ or divorced parents (Table 4). The same can be concluded for educational position. T4-easy-to-recruit participants had attained the highest educational positions at both T2 and T3, whereas T4-non-responders had attained the lowest educational positions at both waves.

**Table 4:** Sociodemographic characteristics of participants who responded to the custom research company hired at the fourth TRAILS measurement wave (T4-easy-to-recruit), participants who responded after extra recruitment effort of the TRAILS research team (T4-hard-to-recruit), participants who did not respond at the fourth wave but did participate in the third wave (non-responder T4) and of participants who had dropped out from TRAILS prior to the fourth wave (drop-out since T2 or T3)

	**T4-easy-to-recruit (ref.)**	** T4-hard-to-recruit**			** Non-responder T4**			** Drop-out since T2 or T3**		
	**n(%)**	** n(%)**	**OR**	**95% CI**	** n(%)**	**OR**	**95% CI**	** n(%)**	**OR**	**95% CI**
*T1 Socio-demographics*										
Girl	894 (55.5)	89 (32.8)	0.39*	0.30-0.51	49 (36.6)	0.46*	0.32-0.67	100 (46.5)	0.70*	0.52-0.93
Non-western immigrant	122 (7.6)	36 (13.3)	1.87*	1.26-2.78	29 (21.6)	3.37*	2.15-5.29	50 (23.3)	3.70*	2.56-5.33
Parents divorced	304 (18.9)	72 (26.6)	1.55*	1.16-2.09	49 (36.6)	2.48*	1.71-3.60	51 (23.7)	1.34	0.95-1.87
Single parent	30 (1.9)	6 (2.2)	1.19	0.49-2.89	6 (4.5)	2.47*	1.01-6.04	11 (5.1)	2.84*	1.40-5.75
No siblings	125 (7.9)	30 (11.2)	1.47	0.97-2.25	16 (12.1)	1.61	0.93-2.81	30 (14.9)	2.05*	1.34-3.15
Lower education mother (≤lower tracks of sec. educ.)	503 (32.0)	113 (43.0)	1.60*	1.23-2.09	82 (64.1)	3.78*	2.60-5.51	119 (60.4)	3.24*	2.39-4.39
Lower education father (≤lower tracks of sec. educ.)	395 (28.3)	84 (38.9)	1.61*	1.20-2.17	52 (50.5)	2.58*	1.72-3.86	78 (48.4)	2.38*	1.71-3.31
Low family income (<€1,135)	187 (12.8)	50 (20.2)	1.72*	1.22-2.43	43 (37.1)	4.02*	2.67-6.03	58 (31.9)	3.19*	2.25-4.51
T1 Low SEP (lowest 25%)	315 (19.8)	83 (31.0)	1.81*	1.36-2.41	73 (55.3)	5.00*	3.47-7.19	82 (40.8)	2.78*	2.05-3.78
Low IQ (wisc < 85)	221 (13.8)	61 (22.6)	1.83*	1.33-2.52	51 (38.6)	3.95*	2.70-5.76	55 (25.8)	2.18*	1.56-3.06
*T4 Socio-demographics*										
Lower education mother (≤lower tracks of sec. educ.)	412 (29.3)	86 (40.6)	1.65*	1.22-2.21	-			-		
Lower education father (≤lower tracks of sec. educ.)	316 (25.3)	71 (39.9)	1.96*	1.41-2.71	-			-		
Low family income (<€1,150)	71 (5.6)	20 (11.0)	2.08*	1.23-3.50	-			-		
T4 Low SEP (lowest 25%)	341 (23.4)	78 (35.6)	1.82*	1.34-2.46	-			-		
	Mean (*SD*)	Mean (*SD*)	OR	95% CI	Mean (*SD*)	OR	95% CI	Mean (*SD*)	OR	95% CI
*Educational level (adjusted for age)*										
T2 Educational level	4.13 (1.32)	3.62 (1.28)	0.64*	0.56-0.74	3.07 (1.17)	0.42*	0.34-0.52	3.42 (1.27)	0.57*	0.48-0.68
T3 Educational level	7.11 (1.57)	6.46 (1.43)	0.61*	0.52-0.73	5.77 (1.28)	0.35*	0.28-0.44	-		

*p < .05; Odds ratios (OR) and 95% confidence intervals (CI) from single multinomial logistic regression analyses (T1, T2 and T3 predictors) and single binary logistic regression analyses (T4 predictors); SEP = Socioeconomic Position; educational level was measured on a 0 – 10 scale, but was standardized for regression analyses; regression analyses with educational level adjusted for age; *n* varies between 1,874 and 2,230 (T1 socio-demographics), 1,426 and 1,617 (T4 socio-demographics), and 1,986 (T2 and T3 educational level).

#### Sociometric status

At T1, being nominated as popular by peers predicted being a responder, whereas being rejected predicted being hard-to-recruit [5]. Peer acceptance at T1 did not predict participation anymore at T4, whereas being rejected by peers, as well as bullying, at T1 still predicted being hard-to-recruit at T4 (Table 5). Hard-to-recruit participants, non-responders, and drop-outs did not differ with respect to being rejected at T1. Thus, sociometric status at T1 differentially predicted participation in a school-based survey at age 11 compared to participation in a web-based survey at age 19.

**Table 5:** Sociometric characteristics of participants who responded to the custom research company hired at the fourth TRAILS measurement wave (T4-easy-to-recruit), participants who responded after extra recruitment effort of the TRAILS research team (T4-hard-to-recruit), participants who did not respond at the fourth wave but did participate in the third wave (non-responder T4) and of participants who had dropped out from TRAILS prior to the fourth wave (drop-out since T2 or T3)

	** T4-easy-to-recruit (ref.)**	** T4-hard-to-recruit**			** Non-responder T4**			** Drop-out since T2 or T3**		
	**Mean (*SD*)**	**Mean (*SD*)**	**OR**	**95% CI**	**Mean (*SD*)**	**OR**	**95% CI**	**Mean (*SD*)**	**OR**	**95% CI**
*T1 Sociometric Characteristics*										
Peer acceptance	29 (15)	27 (17)	0.88	0.72-1.08	26 (16)	0.80	0.60-1.06	27 (16)	0.87	0.71-1.07
Peer rejection	11 (12)	15 (15)	1.29*	1.07-1.56	18 (16)	1.50*	1.19-1.88	16 (16)	1.35*	1.13-1.63
Bullying	05 (07)	07 (09)	1.35*	1.13-1.62	10 (13)	1.56*	1.27-1.93	08 (11)	1.39*	1.16-1.66
Victimization	04 (07)	04 (08)	1.05	0.86-1.28	05 (08)	1.16	0.93-1.37	05 (10)	1.15	0.97-1.37
Helping	22 (14)	19 (14)	0.79*	0.64-0.98	18 (12)	0.75	0.55-1.01	22 (15)	0.97	0.79-1.18
*T2 Sociometric Characteristics*										
Peer acceptance	21 (12)	22 (14)	1.13	0.93-1.38	27 (14)	1.61*	1.25-2.06	20 (13)	0.97	0.75-1.25
Peer rejection	11 (13)	13 (18)	1.15	0.95-1.39	12 (14)	1.09	0.83-1.45	17 (15)	1.44*	1.19-1.75
Bullying	02 (04)	03 (08)	1.45*	1.19-1.76	05 (11)	1.64*	1.32-2.03	05 (09)	1.66*	1.37-2.02
Victimization	02 (05)	03 (08)	1.26*	1.06-1.50	05 (09)	1.48*	1.22-1.79	03 (06)	1.21	0.97-1.50
Helping	25 (13)	22 (14)	0.76*	0.61-0.94	27 (13)	1.18	0.90-1.54	20 (10)	0.68*	0.52-0.89

*p < .05; *p < .05; Odds ratios (OR) and 95% confidence intervals (CI) from single multinomial logistic regression analyses, variables standardized in regression analyses; *n* = 1,065 (T1 sociometrics) and 1,007 (T2 sociometrics). Sociometric status is given in percentages, which were standardized for regression analyses.

Peer acceptance at T2 predicted being a non-responder at T4, while there was no association with being hard-to-recruit or a dropout since T2/T3. Bullying or being a victim of bullying behaviour both predicted being T4-hard-to-recruit, whereas being nominated as a helper predicted being T4-easy-to-recruit.

#### Substance use

Respondents who were easy-to-recruit at T4 were less likely to have used cigarettes or cannabis at T2 than T4-hard-to-recruit participants and T4-non-responders (Table 6). T4-hard-to-recruit participants were more likely than all other groups to have used cannabis at T2, but not at later waves.

**Table 6:** Substance use of participants who responded to the custom research company hired at the fourth TRAILS measurement wave (T4-easy-to-recruit), participants who responded after extra recruitment effort of the TRAILS research team (T4-hard-to-recruit), participants who did not respond at the fourth wave but did participate in the third wave (non-responder T4) and of participants who had dropped out from TRAILS prior to the fourth wave (drop-out since T2 or T3)

	**T4-easy-to-recruit (ref.)**	**T4-hard-to-recruit**			**Non-responder T4**			**Drop-out since T2 or T3**		
	**n(%)**	**n(%)**	**OR**	**95% CI**	**n(%)**	**OR**	**95% CI**	**n(%)**	**OR**	**95% CI**
*Lifetime substance use*										
T2 Tobacco lifetime prevalence	533 (33.7)	107 (44.0)	1.55*	1.18-2.03	62 (50.8)	2.03*	1.40-2.94	56 (39.7)	1.30	0.91-1.84
T2 Alcohol lifetime prevalence	1,162 (73.9)	194 (79.8)	1.40*	1.00-1.95	92 (76.7)	1.16	0.75-1.80	103 (72.5)	0.93	0.64-1.37
T2 Cannabis lifetime prevalence	100 (6.3)	28 (11.6)	1.94*	1.25-3.02	11 (9.3)	1.52	0.79-2.92	14 (10.1)	1.65	0.92-2.98
T3 Tobacco lifetime prevalence	819 (57.1)	89 (70.6)	1.81*	1.21-2.69	69 (70.4)	1.79*	1.14-2.79	-		
T3 Alcohol lifetime prevalence	1,345 (93.9)	125 (98.4)	4.04	0.98-16.62	92 (93.9)	0.99	0.42-2.33	-		
T3 Cannabis lifetime prevalence	439 (30.7)	45 (35.4)	1.24	0.85-1.81	35 (36.5)	1.29	0.84-1.99	-		
T4 Tobacco lifetime prevalence	1,153 (72.7)	100 (81.3)	1.64*	1.03-2.61	-			-		
T4 Alcohol lifetime prevalence	1,146 (98.2)	121 (99.2)	2.27	0.31-16.81	-			-		
T4 Cannabis lifetime prevalence	841 (53.1)	66 (55.0)	1.08	0.75-1.57	-			-		

*p < .05; Odds ratios (OR) and 95% confidence intervals (CI) from logistic regression analyses; *n* varies between 2,074 and 2,087 (T2), 1,651 and 1,658 (T3), and 1,697 and 1,710 (T4).

#### Psychopathology

In terms of externalising problems, the parents of T4-hard-to-recruit participants reported more externalising problems from T1 up to T3 (Table 7). Differences in parent-reported externalising problems between T4 non-responders and drop-outs since T2 or T3 seemed to have diminished over time, whereas differences in self-reported externalising problems emerged at T3 and remained at T4. Hard-to-recruit participants were also more likely to receive a lifetime externalising diagnosis in the CIDI interview at T4. Notably, T4-easy- and hard-to-recruit participants did not differ with regard to self-reported externalising problems at T4. Furthermore, T4-easy-to-recruit participants reported more internalising problems both at T1 and at T3 (Table 7).

**Table 7:** Psychopathology of participants who responded to the custom research company hired at the fourth TRAILS measurement wave (T4-easy-to-recruit), participants who responded after extra recruitment effort of the TRAILS research team (T4-hard-to-recruit), participants who did not respond at the fourth wave but did participate in the third wave (non-responder T4) and of participants who had dropped out from TRAILS prior to the fourth wave (drop-out since T2 or T3)

	** T4-easy-to-recruit (ref.)**	** T4-hard-to-recruit**			** Non-responder T4**			** Drop-out since T2 or T3**		
	**Mean (*SD*)**	**Mean (*SDs*)**	**OR**	**95% CI**	**Mean (*SD*)**	**OR**	**95% CI**	**Mean (*SD*)**	**OR**	**95% CI**
*Psychopathology*										
T1 CBCL Int.	0.24 (0.19)	0.24 (0.21)	0.97	0.85-1.12	0.24 (0.19)	0.97	0.80-1.17	0.27 (0.21)	1.11	0.96-1.29
T1 CBCL Ext	0.22 (0.19)	0.29 (0.21)	1.37*	1.21-1.56	0.31 (0.26)	1.46*	1.24-1.71	0.29 (0.24)	1.36*	1.18-1.57
T1 CBCL Total	0.24 (0.16)	0.27 (0.18)	1.22*	1.07-1.39	0.28 (0.20)	1.27*	1.07-1.50	0.28 (0.19)	1.30*	1.13-1.51
T2 CBCL Int	0.20 (0.18)	0.19 (0.17)	0.95	0.82-1.10	0.20 (0.18)	1.02	0.83-1.25	0.22 (0.21)	1.15	0.97-1.37
T2 CBCL Ext	0.16 (0.18)	0.21 (0.21)	1.30*	1.14-1.47	0.22 (0.27)	1.35*	1.13-1.60	0.20 (0.20)	1.23*	1.03-1.46
T2 CBCL Total	0.18 (0.15)	0.20 (0.16)	1.16*	1.02-1.33	0.21 (0.18)	1.24*	1.03-1.49	0.21 (0.17)	1.22*	1.02-1.45
T3 CBCL Int	0.19 (0.19)	0.21 (0.20)	1.11	0.94-1.32	0.17 (0.18)	0.89	0.67-1.19	-		
T3 CBCL Ext	0.16 (0.18)	0.27 (0.27)	1.49*	1.29-1.72	0.20 (0.24)	1.19	0.94-1.50	-		
T3 CBCL Total	0.17 (0.15)	0.23 (0.19)	1.39*	1.19-1.62	0.19 (0.19)	1.14	0.90-1.46	-		
T1 YSR Int	0.37 (0.24	0.33 (0.21)	0.84*	0.73-0.96	0.34 (0.24)	0.87	0.72-1.05	0.36 (0.26)	0.94	0.81-1.09
T1 YSR Ext	0.27 (0.19)	0.28 (0.20)	1.07	0.94-1.21	0.27 (0.22)	1.03	0.86-1.23	0.29 (0.21)	1.10	0.95-1.26
T1 YSR Total	0.34 (0.19)	0.33 (0.18)	0.92	0.80-1.05	0.33 (0.19)	0.95	0.79-1.14	0.34 (0.21)	0.99	0.86-1.14
T2 YSR Int	0.34 (0.24)	0.29 (0.24)	0.82*	0.71-0.95	0.30 (0.25)	0.83	0.68-1.02	0.30 (0.26)	0.85	0.71-1.03
T2 YSR Ext	0.28 (0.19)	0.31 (0.21)	1.15*	1.01-1.31	0.30 (0.23)	1.09	0.91-1.31	0.28 (0.23)	1.01	0.85-1.21
T2 YSR Total	0.33 (0.18)	0.32 (0.19)	0.96	0.84-1.11	0.31 (0.19)	0.90	0.74-1.09	0.31 (0.20)	0.87	0.73-1.05
T3 YSR Int	0.32 (0.25)	0.26 (0.23)	0.75*	0.60-0.92	0.30 (0.25)	0.94	0.76-1.16	-		
T3 YSR Ext	0.31 (0.21)	0.36 (0.22)	1.25*	1.06-1.48	0.38 (0.25)	1.36*	1.13-1.64	-		
T3 YSR Total	0.33 (0.18)	0.32 (0.17)	0.94	0.78-1.14	0.35 (0.18)	1.10	0.90-1.35	-		
T4 ASR Int	0.25 (0.25)	0.26 (0.27)	1.04	0.86-1.26	-			-		
T4 ASR Ext	0.23 (0.21)	0.25 (0.24)	1.09	0.87-1.36	-			-		
T4 ASR Total	0.28 (0.21)	0.31 (0.25)	1.15	0.92-1.43	-			-		
	n(%)	n(%)	OR	95% CI						
CIDI T4: Int. diagnosis	646 (42.9)	37 (48.1)	1.30	0.78-1.95	-			-		
CIDI T4: Ext. diagnosis	540 (35.8)	39 (50.6)	1.84*	1.16-2.91	-			-		
CIDI T4: Total problem diagnosis	917 (60.8)	53 (68.8)	1.42	0.87-2.33	-			-		

*p < .05; Odds ratios (OR) and 95% confidence intervals (CI) from single multinomial logistic regression analyses (T1, T2 and T3 predictors) and single binary logistic regression analyses (T4 predictors); CBCL = Child Behavior Checklist (parent report); YSR = Youth Self Report; ASR = Adult Self Report; CBCL, YSR and ASR were assessed on a 0–2 scale, but were standardized for analyses. CIDI = Composite International Diagnostic Interview; Int = Internalizing; Ext = Externalizing; continuous variables (CBCL, YSR, ASR) standardized in regression analyses; *n* varies between 2,044 and 2,055 (T1 CBCL), 1,901 and 1,924 (T2 CBCL), 1,494 and 1,507 (T3 CBCL), 2,170 and 2,188 (T1 YSR), 2,081 and 2,092 (T2 YSR), 1,636 and 1,660 (T3 YSR), 1,654 and 1,691 (T4 ASR), and 1,584 (T4 CIDI).

In the current analysis, with T4-easy-to-recruit participants as reference category, we cannot show whether T4-non-responders differ significantly from T4-hard-to-recruit participants. Results from the analysis with T4-hard-to-recruit participants as reference category show that T4-non-responders significantly more often have a low educated mother, low family income, low SEP, low IQ and lower educational position compared to T4-hard-to-recruit responders. In terms of psychopathology, substance use and other sociodemographic variables, the differences were not statistically significant (results not shown but available upon request).

Finally, the multiple regression analysis shows that being T1-hard-to-recruit most strongly predicts recruitment status at T4, and furthermore that being male, from non-Western origin, having a low educated mother, low family income, low IQ and having internalising and externalising problems remain statistically significant risk factors for being T4-hard-to-recruit in a multivariate model (Table 8). Analyses including interaction terms yielded strong main effects of both recruitment status and predictors; their interaction however yielded negligible effects in the opposite direction. These interaction results might be unreliable resulting from the small numbers in the various categories.

**Table 8:** Multiple multinomial logistic regression analysis predicting T4-easy-to-recruit, T4-hard-to-recruit, T4-non-responder, and drop-out since T2 or T3

	**T4-easy-to-recruit **(*n* = 1,359) (ref.)****	**T4-hard-to-recruit **(*n* = 213)****			**T4-non-responder **(*n* = 98)****			**Drop-out since T2 or T3 **(*n* = 150)****		
	**n(%)**	**n(%)**	**OR**	**95% CI**	**n(%)**	**OR**	**95% CI**	**n(%)**	**OR**	**95% CI**
*T1 Recruitment Effort*										
Hard to recruit	42 (3.1)	8 (3.8)	0.98	0.44-2.17	13 (13.3)	2.51*	1.21-5.18	18 (12.0)	2.65*	1.42-4.96
*(univariate effects)*			*1.22*	*0.57-2.64*		4.80*	*2.48-9.28*		4.28*	*2.39-7.64*
*T1 Socio-demographics*										
Girl	747 (55.0)	66 (31.0)	0.41*	0.30-0.56	40 (40.8)	0.64*	0.41-1.00	72 (48.0)	0.84	0.59-1.20
*(univariate effects)*			0.37*	*0.27-0.50*		0.57*	*0.37-0.56*		*0.76*	*0.54-1.06*
Non-western immigrant	87 (6.4)	23 (10.8)	1.66	1.00-2.76	23 (23.5)	3.11*	1.76-5.47	29 (19.3)	2.67*	1.62-4.39
*(univariate effects)*			1.77*	*1.09-2.87*		4.48*	*2.68-7.51*		3.50*	*2.21-5.55*
Parents divorced	248 (18.2)	33 (26.8)	1.45	0.99-2.12	33 (33.7)	1.47	0.88-2.47	32 (21.3)	0.81	0.51-1.31
*(univariate effects)*			1.64*	*1.17-2.28*		2.27*	*1.46-3.54*		*1.22*	*0.80-1.84*
Lower educ.mother (≤lower tracks of sec. educ.)	422 (31.1)	88 (41.3)	1.30	0.94-1.78	60 (61.2)	2.24*	1.42-3.54	88 (58.7)	2.52*	1.75-3.64
*(univariate effects)*			1.56*	*1.16-2.10*		3.51*	*2.30-5.35*		3.15*	*2.23-4.45*
Low family income (<€1,135)	157 (11.6)	40 (18.8)	1.23	0.78-1.92	34 (34.7)	1.88*	1.09-3.24	38 (25.3)	1.63*	1.01-2.64
*(univariate effects)*			1.77*	*1.21-2.60*		4.07*	*2.60-6.37*		2.60*	*1.74-3.89*
Low IQ (wisc < 85)	180 (13.2)	44 (20.7)	1.43	0.96-2.12	37 (37.8)	2.27*	1.40-3.67	39 (26.0)	1.43	0.93-2.20
*(univariate effects)*			1.71*	*1.18-2.46*		3.97*	*2.57-6.15*		2.30*	*1.55-3.42*
*T1 Psycho-pathology*	mean (*SD*)	mean (*SD*)	OR	95% CI	mean (*SD*)	OR	95% CI	mean (*SD*)	OR	95% CI
CBCL Int	0.25 (0.19)	0.24 (0.21)	0.81*	0.67-0.98	0.25 (0.20)	0.75*	0.58-0.97	0.26 (0.21)	0.96	0.78-1.18
*(univariate effects)*			*0.97*	*0.84-1.12*		*1.00*	*0.81-1.23*		*1.09*	*0.92-1.28*
CBCL Ext	0.22 (0.19)	0.29 (0.21)	1.38*	1.17-1.62	0.31 (0.27)	1.43*	1.14-1.79	0.29 (0.24)	1.29*	1.06-1.56
*(univariate effects)*			1.38*	*1.21-1.57*		1.48*	*1.24-1.76*		1.34*	*1.15-1.57*
YSR Int	0.37 (0.24)	0.33 (0.21)	0.88	0.75-1.03	0.36 (0.25)	0.88	0.71-1.10	0.33 (0.24)	0.77*	0.64-0.94
*(univariate effects)*			0.85*	*0.73-0.99*		*0.96*	*0.78-1.17*		*0.85*	*0.71-1.02*

*p < .05; Odds ratios (OR) and 95% confidence intervals (CI) from multivariate multinomial logistic regression analysis; CBCL = Child Behavior Checklist (parent report); YRS = Youth Self Report; Int = Internalizing; Ext = Externalizing; CBCL and YSR scores standardized; univariate effects of single multinomial regression analyses italicized. Nagelkerke *R²* = 0.159.

## Discussion

### Main findings regarding effects of recruitment efforts eight years later

The response rate after eight years follow up is 84%; among the T1 hard-to-recruit participants we found no significant differences between participants and non-participants at T4 in demographic variables, peer status or psychiatric symptoms. This indicates there is no selective attrition of the most vulnerable T1-hard-to-recruit participants along the four measurement waves. We may conclude that extensive recruitment effort does not only increase the representativeness of the sample at initial assessment waves [5,11,12], but also eight years later. This is an important finding. We encourage other researchers to investigate retention rates of easy-to-recruit and hard-to-recruit participants in their longitudinal samples to examine the robustness of these findings.

A response rate of 84% at eight year follow-up can be considered high. Although response rates in some other studies are unequalled [44], reported response rates are usually similar [15,18,45] or lower in population-based cohorts [13,14,17,19]. Two population-based studies have reported eight year follow-up rates [19,45]. In the Great Smoky Mountains Study (GSMS), the initial inclusion rate was 80%, and the participation rate after eight years follow-up ranged from 77-83% in three different cohorts [45], giving a total response rate of about 62-66%. Total response rates of the Avon Longitudinal Study of Parents And Children (ALSPAC) seem somewhat lower, that is, 54% after eight years follow-up [19]. The total response rate in TRAILS was 64% after eight years. Total response rates in population studies in which participants with a certain psychiatric disorder are oversampled are usually remarkably lower. For example, the Netherlands Study of Depression and Anxiety or NESDA achieved a two-year follow-up response rate of 87%, but the initial response rates were low. Less than 50% of individuals recruited through primary care or from other cohort studies, and 57% of patients recruited via specialized mental health care settings enrolled in the study [46], giving a total response of about 44%.

### Main findings regarding effects of extensive recruitment efforts at age 19

#### Sociodemographic characteristics

Like at T1 [5], we can conclude that, although differences between participants (T4-easy and hard-to-recruit) and non-participants (T4-non-responders and drop-outs since T2/T3) on sociodemographic variables decreased, they did not disappear with extensive recruitment efforts. This conclusion parallels conclusions from other studies that sociodemographic variables predict being hard-to-recruit [11,12] and non-response [3,13-18].

#### Sociometric status

As far as we know, the association between peer nominations for sociometric status and response or attrition has not been studied in other samples than TRAILS [5]. At T1, being nominated as popular by peers predicted being a T1- responder, whereas being rejected predicted being T1-hard-to-recruit [5]. However, peer acceptance at T1 did not predict recruitment status at T4, while peer rejection, as well as bullying and being bullied still predicted being T4-hard-to-recruit. We might speculate that popular children felt encouraged to participate in a school-based survey, whereas this type of positive peer pressure did not influence their decision to participate eight years later in a web-based survey. Peer rejection, bullying and being bullied at T1 however remain important predictors for being hard-to-recruit, also 8 years later in a web-based survey. It would be interesting to investigate how peer acceptance or rejection predicted participation rates in cohort studies that used simultaneous school and web-based surveys in the same age groups [13,14].

#### Substance use

Substance use has been shown to be a predictor of being hard-to-recruit, being a non-responder or dropping out at follow-up [12,13,18,47]. Indeed, hard to recruit respondents were more likely to have used alcohol, cannabis and cigarettes. The fact that T4-hard-to-recruit responders reported more cannabis use at T2 suggests that the extensive recruitment efforts at T4 increased representativeness of the whole sample.

#### Psychopathology

The finding that parent-reported problems decreased over time while self-reported problems seemed to emerge could be related to the decreasing knowledge the parent has of the behaviour of the child as the child grows older. That easy- and hard-to-recruit participants did not differ with regard to self-reported externalizing problems at T4 might indicate that the effect of extensive recruitment efforts at T4 increased the number of participants high on externalizing behaviours, like it did at T1 [5]. Indeed, subjects high on externalizing problems have been shown to be less likely to respond to single recruitment efforts [11,14] and more likely to drop-out from longitudinal studies [13,15,19]. Extensive recruitment efforts at age 11 also decreased differences between participants and non-participants on internalizing problems [5]: teachers reported more internalizing problems for T1-hard-to-recruit participants than for T1-easy-to-recruit participants. At age 19, there seems to be a different trend*.* Easy-to-recruit participants at T4 reported more internalizing problems both at T1 and at T3 (Table 4). This might have been a report bias as these differences were not apparent in parent-reported internalizing problems, nor were T4 easy-to-recruit participants more likely to have received a lifetime internalizing diagnosis in the CIDI interview at T4. Results from other studies are inconsistent with respect to internalizing problems as well; whereas most found that internalizing problems did not predict response [6,11,13,15,16], others showed that individuals with internalizing problems were less likely to participate [14] or more likely to drop-out at follow-up [46].

Overall, we conclude that the extra recruitment efforts of the TRAILS research team have increased the number of vulnerable adolescents participating in the fourth wave over and above the recruitment efforts of the CRC, resulting in a similarly diverse sample that was reached by the extensive recruitments efforts at T1, giving confidence in estimated associations in TRAILS studies.

### Limitations

In spite of intensive recruitment efforts we were not able to contact all T4-non-responding TRAILS participants. This means we have no information about their current (mental) health status, substance use or educational level. Also, at T2 and T3, we did not contact non-responders to collect reasons for non-response or information regarding their current (mental) health status and other measures. Therefore, information on factors predicting non-response at T3 and T4 is derived from earlier measurement waves in which the non-responders still participated.

Furthermore, the measurement of sociometric status was only possible in classrooms with at least 10 TRAILS participants [35]. This lead to a much smaller number of participants for these measures (at T1 N = 1065; at T2 N = 1023 for the peer nominations).

### Implications of the findings

The results that are presented here have implications in two fields. First, when setting up a longitudinal study, researchers might want to put extra effort in recruiting initial non-responders as we have shown this pays off in the short and long term. It results in enrolling a more representative sample at baseline, and ensures increased generalisability even after eight years and four assessment waves later, when over 60% of those who were hard-to-recruit at baseline are still in the sample. We found that there are no significant differences between T1-hard-to-recruit dropouts and T1-hard-to-recruit retainers in terms of sociodemographic variables, peer status or psychiatric symptoms, indicating we did not lose the most vulnerable T1-hard-to-recruit participants and the increased generalisability of the sample is maintained.

Second, the results of this paper might have implications for the analysis of longitudinal data, wherein researchers are commonly confronted with missing data. Missing values can be dealt with by multiple imputation, which has been shown to cause less bias compared to complete case analysis, single imputation or the missing indicator method [48]. Based on the results presented in this paper, ‘drop out’ could be modelled, which might aid researchers in decisions they need to make when imputing data for missing participants or participants with missing data.

## Conclusions

First, we conclude that extensive recruitment efforts at the first assessment wave of a population-based cohort still pays off eight years later. Over 60% of T1 hard-to-recruit responders who were persuaded to participate by extensive recruitment efforts still participated in the study four assessment waves later. This is an important conclusion, especially for researchers who are designing a population-based cohort study and have to decide whether or not to invest in recruiting initial non-responders.

Second, we conclude that the effects of extensive recruitment effort are largely similar in different age groups using different survey methods. Differences between easy and hard-to-recruit responders at the first assessment wave, when the mean age was 11 and a school-based assessment method was used, were very similar to the differences between easy and hard-to-recruit responders at the fourth wave, when the mean age was 19 and a web-based survey method was used. At both measurement waves, differences between responders and non-responders decreased after inclusion of hard-to-recruit participants.

## Competing interests

FCV is a contributing author of the Achenbach System of Empirically Based Assessment, from which he receives remuneration. All other authors declare that they had no competing interests.

## Authors' contributions

FJ and EN are responsible for the development and design of the study. DR performed the statistical analysis in close collaboration with FJ and EN. FJ and EN wrote the first draft. JO and AJO were involved in the interpretation of the results. RV is responsible for the construction of the measure of educational position. JO, AJO, RV and FCV provided critical comments on earlier versions of the paper. All authors had full access to all of the data (including statistical reports and tables) in the study and can take responsibility for the integrity of the data and the accuracy of the data analysis. All authors approved the final submitted version.

## Data Sharing

TRAILS data of the T1 and T2 measurement waves are deposited in DANS-KNAW and can be accessed at http://www.dans.knaw.nl.

## Pre-publication history

The pre-publication history for this paper can be accessed here:

http://www.biomedcentral.com/1471-2288/12/93/prepub
